# Cytotoxic and genotoxic evaluation of cotinine using human neuroblastoma cells (SH-SY5Y)

**DOI:** 10.1590/1678-4685-GMB-2019-0123

**Published:** 2020-05-29

**Authors:** Daiana Dalberto, Caroline Cardoso Nicolau, Ana Leticia Hilario Garcia, Adriane Perachi Nordin, Ivana Grivicich, Juliana da Silva

**Affiliations:** 1 Universidade Luterana do Brasil (ULBRA), Programa de Pós-Graduação em Biologia Celular e Molecular Aplicada à Saúde - PPGBioSaúde, Laboratório de Toxicologia Genética, Canoas, RS, Brazil; 2 Universidade Feevale, Programa de Pós-Graduação em Qualidade Ambiental, Laboratório de Ecotoxicologia, Novo Hamburgo, RS, Brazil; 3 Universidade Luterana do Brasil (ULBRA), Programa de Pós-Graduação em Biologia Celular e Molecular Aplicada à Saúde - PPGBioSaúde, , Laboratório de Biologia de Cancer, Canoas, RS, Brazil; 4 Universidade La Salle, Programa de Pós-Graduação em Saúde e Desenvolvimento Humano, Canoas, RS, Brazil

**Keywords:** Cotinine, nicotine, cytotoxicity, genotoxicity, SH-SY5Y cells

## Abstract

Cotinine is the main metabolite of nicotine, which is metabolized in the liver through a cytochrome P450 enzyme. Different studies point to genetic instability caused by nicotine, such as single and double DNA strand breaks and micronuclei formation, but little is known about the effect of cotinine. Therefore, the present *in vitro* study assessed the effects of cotinine on cell viability and DNA damage in SH-SY5Y neuroblastoma cells, as well as genotoxicity related to oxidative stress mechanisms. Comparisons with nicotine were also performed. An alkaline comet assay modified by repair endonucleases (FPG, OGG1, and Endo III) was used to detect oxidized nucleobases. SH-SY5Y neuronal cells were cultured under standard conditions and exposed for 3 h to different concentrations of cotinine and nicotine. Cytotoxicity was observed at higher doses of cotinine and nicotine in the MTT assay. In the trypan blue assay, cells showed viability above 80% for both compounds. Alkaline comet assay results demonstrated a significant increase in damage index and frequency for cells treated with cotinine and nicotine, presenting genotoxicity. The results of the enzyme-modified comet assay suggest a DNA oxidative damage induced by nicotine. Unlike other studies, our results demonstrated genotoxicity induced by both cotinine and nicotine. The similar effects observed for these two pyridine alkaloids may be due to the similarity of their structures.

## Introduction

**Figure 1:**
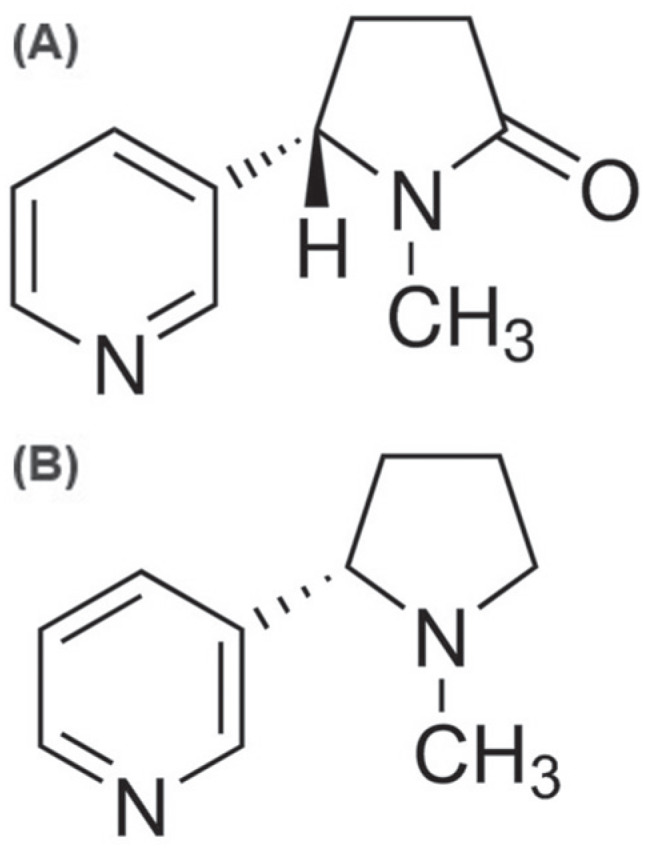
Chemical structure of cotinine (A) and nicotine (B).

Nicotine is an alkaloid found in tobacco leaves (*Nicotiana tabacum*), a plant native from the Americas (Schroff *et al.*, 2000; El-Sakka, 2010; Lee *et al.*, 2015; Sinditabaco, 2017). The metabolism of nicotine occurs mainly in the liver through cytochrome P450 enzymes, mainly CYP2A6. Approximately 70% to 80% of nicotine is metabolized into cotinine, an alkaloid considered toxic (Hukkanen *et al.*, 2005; Henningfield *et al.*, 2009). There is evidence that cotinine may lead to the same effects as nicotine, probably due to structural similarity (Figure 1) (Grizzell and Echeverria, 2015).

Agricultural workers engaged in tobacco cultivation are constantly exposed to nicotine, which is present in the plant leaves. According to Da Silva *et al.* (2010), Fassa *et al.* (2014), and Mishra *et al.* (2015), the tobacco producers who have contact with tobacco leaves at the harvest period, often humid, are at risk of intoxication. This acute nicotine intoxication is known as Green Tobacco Sickness (GTS), and is caused by transdermal absorption of the substance. Nicotine also enters the bloodstream almost instantly and crosses the blood-brain barrier within seconds (Luttrell and Vogel, 2014; Raja, 2016), thus affecting human behavior, central and peripheral nervous systems, and cardiovascular and endocrine systems, among other problems (Sassone, 2011).

GTS is only related with nicotine. Although the action of cotinine in humans is still poorly understood, it is known to have a longer biological half-life than nicotine. Nicotine is rapidly removed from the central nervous system (1–3 hours), while cotinine remains in the organism for a longer period (10–30 hours) (Sassone, 2011; Katner *et al.*, 2015). The accumulation of high concentrations of cotinine in the brain, along with its pharmacological potential, suggests that cotinine should be examined for its possible involvement in the nicotine effect and dependence (Katner, 2015).

Different studies have tested potential carcinogenic effects of cotinine, but evidence is inconclusive, due to the lack of studies with adequate desing and conflicting or incomplete results (Haussmann and Fariss, 2016). Although there is also no evidence that nicotine alone causes cancer (Luttrell and Vogel, 2014), different studies using different experimental designs point to genetic instability caused by nicotine (Kleinsasser *et al.*, 2005; Attia, 2007; Sobkowiak and Lesicki, 2009; Da Silva *et al.*, 2010; Lomazzo *et al.*, 2011; Nishioka *et al.*, 2011; Ginzkey *et al.*, 2012, 2013, 2014; Kahl *et al.*, 2012; Gao *et al.*, 2014; Sanner and Grimsrud, 2015).

Human neuroblastoma cell lines are commonly used in studies related to neurotoxicity, oxidative stress, and neurodegenerative diseases (Krishna *et al.*, 2014). These cell lines are used in *in vitro* experiments that require neuron-like cells (Kovalevich and Langfor, 2013). Therefore, this study aimed to evaluate cytotoxic and genotoxic effects, and to detect oxidative stress caused by different concentrations of nicotine and cotinine through the modified comet assay using the human neuroblastoma cell line SH-SY5Y.

## Material and Methods

### Cell culture

Human neuroblastoma cells (SH-SY5Y) were purchased from Rio de Janeiro Cell Bank (BCRJ, Duque de Caxias, RJ, Brazil). The cell line was established in 1970 from a metastatic bone tumor. Neuroblastoma (NB) derived cell lines carry the wild-type p53 gene with a p53-dependent apoptotic pathway.

Cell cultures were maintained under specific standard conditions in humid atmosphere at 37 °C and 5% CO_2_, in DMEM/F12 medium (Dulbecco's Modified Eagle's Medium/Ham's Nutrient Mixture F12) supplemented with fetal bovine serum (FBS) (10%) and stabilized (1%) antibiotic antimycotic solution (100X).

For the MTT [3-(4,5-dimethyl-2-thiazolyl)-2,5-diphenyl-2H-tetrazolium bromide], trypan blue, alkaline comet, and modified comet assays, cells (1 × 10^5^) were seeded in complete medium and cultured in 24-well plates to allow cell adhesion. The determination of the concentrations of nicotine and cotinine followed the International Standard ISO/EN10993-5 (2009). In each well, the concentrations of cotinine (2.0, 1.0, 0.5, 0.25, and 0.125 mg/mL) and nicotine (2.0, 1.0, 0.5, 0.25, 0.125 μL/mL) were added, as well as DMEM/F12 medium as a negative control and dimethyl sulfoxide (DMSO 20%) or hydrogen peroxide (H_2_O_2_ 2 mM) as a positive control. For the modified comet assay, only the concentrations of 2.0, 0.5, and 0.125 mg/mL for cotinine and 2.0, 0.5, and 0.125 μL/mL for nicotine were used. Compounds were purchased from Sigma-Aldrich. The culture medium DMEM/F12 was used for the dilution of the compounds.

### Cell viability assay

To determine the cytotoxicity, the colorimetric assay of MTT was performed following the International Standard ISO/EN10993-5 (2009) and Mosmann (1983), with minor modifications.

After the treatments, the cells were washed with DPBS, incubated with 150 μL/well of MTT solution (1 mg/mL in DPBS) in phenol-free culture medium at 37 °C for 3 h. After incubation, the supernatant was carefully removed and the violet formazan crystals were solubilized in 100 μL DMSO.

The absorbance reading of the formazan crystals, which is directly proportional to the number of viable cells, was performed using an ELISA reader with a wavelength of 540 nm (Multiskan, UNISCIENCE). The tests were performed in duplicate.

The trypan blue method is widely used to evaluate cytotoxicity in experimental investigations (Avelar-Freitas *et al.*, 2014). The principle of the assay is that living cells have an intact plasma membrane that prevents the internalization of dyes, such as trypan blue. The evaluation occurs through the analysis of cells in a hemocytometer, in which the uncolored cells represent the viable cells and the blue-colored are the non-viable cells (Louis and Siegel, 2011). The experiments were performed in duplicate.

In addition to the aforementioned concentrations, the assay had a negative control (DMEM/F12 medium) and a positive control (2 mM H_2_O_2_). After 3 hours of exposure, the cells were trypsinized for 3 min in 5% CO_2_ at 37 °C and centrifuged for 3 min at 1500 rpm. The supernatant was removed and cells were resuspended with culture medium. Cells were then homogenized with 0.4% trypan blue dye in the ratio 1:1 (dye:cell homogenate) and subsequently membrane integrity was assessed by reading in an automated cell counter. The results are reported as the total percentage of viable cells, in both the MTT assay and the trypan blue assay.

### Alkaline comet assay

The cell preparation was done as aforementioned. The negative control used was DMEM/F12 medium and the positive control was 2 mM H_2_O_2_.

The alkaline comet assay was performed as described by Singh *et al.* (1988) and modified by Da Silva *et al.* (2000). After a 3-hour treatment, SH-SY5Y cell suspensions (20 μL) were dissolved in 80 μL of low melting agarose and arranged in microscope slides pre-coated with a layer of 1% normal melting point agarose. After solidification of the mixture the slides were dipped in lysis buffer (2.5 M NaCl, 100 mM EDTA, 10 mM Tris, pH 10.0 - 10.5) containing 1% (v/v) Triton X-100 and 10% (v/v) of DMSO, protected from light.

After 1 hour in the lysis buffer, the slides were placed in horizontal electrophoresis cuvettes and covered with alkaline buffer solution (300 mM NaOH and 1 mM EDTA, pH > 13) prepared at the time of use, at 4 °C for 20 min in order to facilitate DNA unwinding. The DNA was then electrophoresed for 15 min at 25 V (0.90 V/cm) and 300 mA. Subsequently, the slides were neutralized with 0.4 M Tris buffer solution (pH 7.5) and stained with silver nitrate solution.

The analysis was performed on 100 cells per treatment (50 per slide) using a light microscope. Cells were classified into different classes of damage from 0 to 4, being class 0: undamaged, without a tail; class 1: with tail shorter than the diameter of the head (nucleus); class 2: with tail 1–2 times longer than the diameter of the head; class 3: with a tail 2 times longer than the diameter of the head; class 4: significant damage, with a long tail, measuring more than 3 times the diameter of the head. A value (damage index, DI) was assigned to each comet according to its class. DI ranged from 0 (completely undamaged: 100 cells X 0) to 400 (with maximum damage: 100 cells X 4). Each concentration was tested in quadruplicate from two independent experiments.

### Modified comet assay

For the modified comet assay with endonucleases, cells were prepared in the same way as for the conventional comet assay until the slides were removed from the lysis solution.

The modified comet assay was performed according to Collins *et al.* (1993). When the slides were removed from the lysis solution, they were washed three times with buffer solution (400 mM Hepes, 1 M KCl, 5 mM EDTA, 2 mg/mL BSA, pH-8.0) and then incubated at 37 °C with enzyme buffer supplemented with formamidopyrimidine DNA glycosilase (FPG) (1 μg/mL solution), endonuclease III (Endo III) (1 μg/mL solution) for 45 min, and DNA glycosylase (OGG1) (1 μg/mL solution) for 30 min in a humid chamber. After this step, the slides were placed in a horizontal electrophoresis chamber and covered with alkaline buffer solution (300 mM NaOH and 1 mM EDTA, pH > 13) freshly prepared, remaining for 40 min. The DNA was then electrophoresed for 15 min at 25 V (0.90 V/cm) and 300 mA. Neutralization, staining, and damage assessment occurred as described for the conventional alkaline comet. Arbitrary units from the alkaline comet assay (without enzyme) represent the DNA strand breaks. Sensitive sites were calculated by subtracting the arbitrary units of the test without enzyme from the arbitrary units of the enzyme-treated test. The experiments were performed in quadruplicate.

### Statistical analysis

For the statistical analysis between the different concentrations of the samples, the univariate analysis of variance (ANOVA) was used followed by the Kruskal-Wallis or Tukey's test using GraphPad Prism 5.0 software, with a value of *p* < 0.05 considered as indicative of statistical significance.

## Results

**Figure 2:**
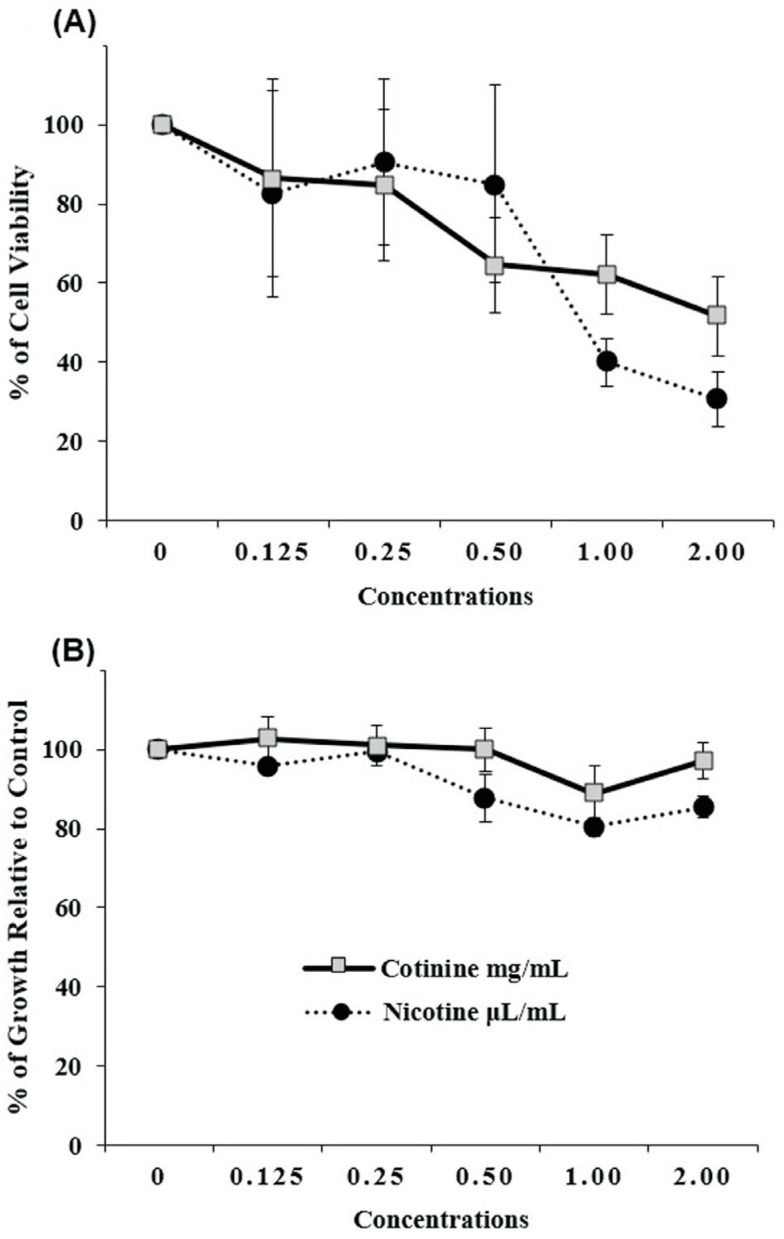
Cell viability evaluation of SH-SY5Y using the MTT assay (A) and using the Trypan Blue assay (B) after cotinine and nicotine treatment (3 h) (mean ± SE). Experiments conducted in quadruplicate.

Results for the MTT assay demonstrated cytotoxicity in neuroblastoma cells above 70% for concentrations greater than 0.25 mg/mL of cotinine and greater than 0.5 μL/mL of nicotine (Figure 2A). For the cell viability assay with trypan blue, cells presented cell viability above 80% for all concentrations, for both cotinine and nicotine, not exhibiting high cytotoxicity (Figure 2B). The results showed no significant difference for MTT and trypan blue results between groups (*p* > 0.05; ANOVA, Tukey's test).

The alkaline comet assay presented significant results for the damage index and frequency, for both cotinine and nicotine compounds. These results demonstrated induction of genotoxicity in human neuroblastoma cells (*p* < 0.05; ANOVA, Kruskal-Wallis test) (Table 1).

**Table 1:** Evaluation of DNA damage in SH-SY5Y cells treated with cotinine and nicotine. Results are reported as mean ± standard deviation.

Groups	Damage Index (0-400)	Damage Frequency (%)
Negative Control	75.82 ± 64.17	41.41 ± 24.77
Cotinine		
0.125 mg/mL	151.25 ± 30.39^b^	67.50 ± 11.85^c^
0.250 mg/mL	176.50 ± 32.14^c^	72.25 ± 9.91^c^
0.500 mg/mL	171.50 ± 46.26^c^	78.00 ± 13.29^c^
1.00 mg/mL	151.50 ± 19.36^b^	74.75 ± 7.04^c^
2.00 mg/mL	201.25 ± 66.77^c^	85.00 ± 11.46^c^
Nicotine		
0.125 μL/mL	171.00 ± 38.93^a^	75.00 ± 17.76^c^
0.250 μL/mL	139.75 ± 15.92^a^	69.00 ± 9.90^c^
0.500 μL/mL	208.25 ± 59.35^b^	87.50 ± 11.45^c^
1.00 μL/mL	250.75 ± 71.62^c^	88.25 ± 10.90^c^
2.00 μL/mL	308.00 ± 88.54^c^	92.50 ± 8.35^c^
Positive Control^d^	253.60 ± 104.38^c^	85.80 ± 20.63^c^

a Statistical significance compared to negative control at *p* < 0.05,

b at *p* < 0.01, and

c at *p* < 0.001) (Kruskal-Wallis test).

d H_2_O_2_ - 2 mM.

**Figure 3:**
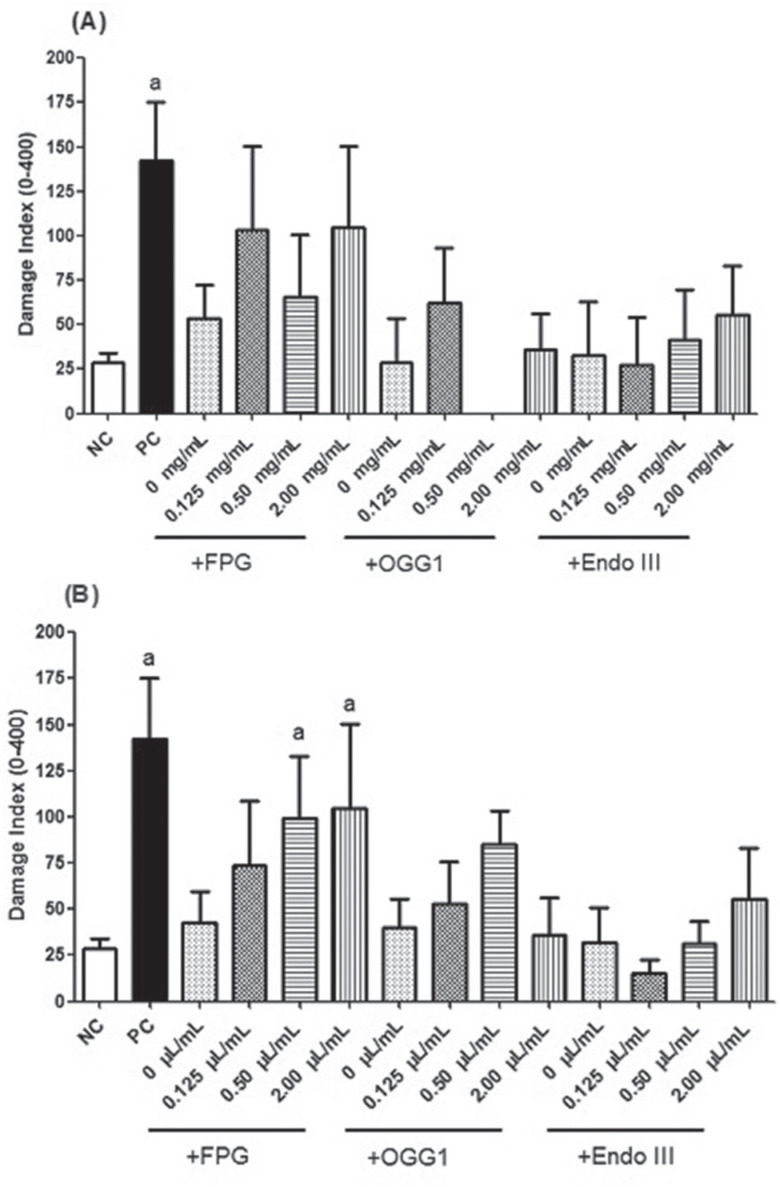
Damage index results (mean ± SD) obtained with modified comet assay using FPG, OGG1 and Endo III sensitive sites on SH-SY5Y cells exposed to cotinine (A) and nicotine (B). ^a^Significant at *p* < 0.05 in relation to negative control (NC; without enzyme= strand breaks) (Kruskal-Wallis test). PC: Positive control (H_2_O_2_: 2 mM; without enzyme). Oxidative DNA damage (cells exposed to enzymes) was calculated as the difference between the scores obtained before and after incubation with the respective enzyme or the buffer.

The modified comet assay (FPG, OGG1, and Endo III) demonstrated no significant increase in total DNA damage in SH-SY5Y cells exposed to cotinine and nicotine (Figure 3) at all concentrations, except at 0.5 and 2 μL/mL of nicotine using FPG (*p* < 0.05; Kruskal-Wallis test).

## Discussion

Studies on the genotoxicity of nicotine are still controversial, and little is known about its major metabolite, cotinine (Wise and Bozarth, 1987). Our study evaluated the cytotoxic and genotoxic effects of cotinine and nicotine in a human neuroblastoma cell line using the alkaline comet assay.

Cytotoxicity was evaluated using two methods, the MTT assay, which evaluates mitochondrial activity through mitochondrial respiration and can be used to assess cellular energy capacity (Mosman, 1983, Meerloo and Cloos, 2011), and the trypan blue assay, which evaluates the integrity of the cell membrane (Avelar-Freitas *et al.*, 2014). Cell viability evaluation is a useful tool for different experimental procedures (Avelar-Freitas *et al.*, 2014). We observed very similar results between cotinine and nicotine in both assays. The MTT results indicated that concentrations of 0.25 and 0.125 mg/mL of cotinine and 0.5, 0.25, and 0.125 μL/mL of nicotine showed viability above 70%, when compared to the negative control (100%), in 3 hours of exposure, indicating that cytotoxicity can reduce cell viability in a dose-dependent manner.

Higher cytotoxicity was found at the highest concentrations of both cotinine (2.0, 1.0, and 0.5 mg/mL) and nicotine (2.0 and 1.0 μL/mL). In the trypan blue assay, low cytotoxicity was observed for the SH-SY5Y cell line, since all concentrations for both compounds showed cell viability > 80%. Similar to our study, other studies have also demonstrated nicotine cytotoxicity in the MTT assay in different cell lines, including human cells (Kang *et al.*, 2011; Zeng *et al.*, 2012). Wang *et al.* (2014) have shown that cell viability was significantly inhibited by nicotine in a dose- and time-dependent manner. The study by Ginzkey *et al.* (2009) evaluated cytotoxicity via the trypan blue test, where cells exposed to nicotine were not harmed, presenting cell viability higher than 80%. Using other tests to evaluate cytotoxicity, such as human fibroblast cells and hepatocellular carcinoma, other authors have also demonstrated similar nicotine and cotinine responses (Babich and Borenfreund, 1992; Esfahrood *et al.*, 2015). In order to evaluate the genotoxicity of cotinine and nicotine in human neuroblastoma cells, an alkaline comet assay was performed. The results of this assay showed genotoxicity in cells exposed to cotinine and nicotine, for damage index and frequency. Higher concentrations of nicotine (1 - 2 μL/mL) induced an increase in DI greater than 3-fold, and cotinine (1 - 2 mg/mL) resulted in an increase greater than two-fold when compared to the negative control. It was not possible to observe a dose-response in the comet assay, possibly because cytotoxicity occurred at higher concentrations as in the MTT test.

The results of the MTT assay indicate that the cell metabolism stopped after exposure to cotinine or nicotine, but no membrane damage was detected, as evidenced by the trypan blue assay. Together with the genotoxicity data, it is likely that DNA damage is so extensive that it killed the cells, explaining the cytotoxic effects. These effects may be explained by oxidative damage due to decreased mitochondrial function induced by cotinine/nicotine, leading to an increase in DNA damage followed by cell death. It is well established that when producing ATP through oxidative phosphorylation, mitochondria also are the major site of intracellular reactive oxygen species (ROS) production. Mitochondria themselves are susceptible to ROS, and injured mitochondria produce higher levels of ROS that cause further mitochondrial dysfunction (Lin and Beal, 2006). The excess of ROS also causes oxidative damage to DNA, proteins, and lipids. In DNA, ROS can provoke single- and double-stranded DNA breaks (Evans *et al.*, 2004),

Data from previous studies already point to DNA damage caused by exposure to nicotine (Ginzkey *et al.*, 2012, 2013, 2014), as demonstrated in our study through the exposure of SH-SY5Y to different concentrations of nicotine. Although different studies in the literature point to the genotoxicity of nicotine, and only few were done for cotinine, it can be inferred that the DNA damage observed *in vivo* in rodents is induced by both alkaloids, since nicotine is mostly metabolized into cotinine (and cotinine remains for a long time in the body). Likewise, Kahl *et al.* (2012) and Da Silva *et al.* (2013) through the comet assay and micronucleus test have demonstrated that nicotine was genotoxic and mutagenic in mice. While the comet assay revealed DNA damage that can be repaired, micronuclei are biomarkers of loss or breaks of entire chromosomes that cannot be repaired. Evidence suggests that exposure to nicotine may interfere with different cellular processes that are considered important for the promotion or progression of carcinogenic processes. Studies have reported that nicotine stimulates cell proliferation, induces cell migration, inhibits apoptosis, induces angiogenesis, and inhibits immune functions. Haussmann and Fariss (2016) reviewed studies related to nicotine and cancer, showing that the majority of studies (∼70%) provide sufficient evidence to conclude that nicotine can stimulate carcinogenesis in animals. The role of nAChRs has been emphasized in the process of triggering intracellular signaling pathways, which in turn influence the carcinogenic process (Haussmann and Fariss, 2016).

As mentioned before, the comet assay is a widely used method for assessing DNA damage and, with modifications, it is possible to evaluate different types of DNA damage. Through improvements, the assay became more sensitive and confirmatory in relation to mechanisms of oxidative damage (Collins, 2014). In order to understand the mechanisms of action of cotinine and nicotine, we used the comet assay modified by repair endonucleases with FPG, OGG1, and Endo III enzymes. The use of the enzymes led to increased DNA damage by both cotinine and nicotine compared to the positive control, but only the highest concentrations of nicotine presented significantly higher results when using the FPG enzyme when compared to the negative control. Through the FPG enzyme, it is possible to detect the presence of altered purines (Langie *et al.*, 2015). Some of the damaged bases that are recognized and removed by FPG include 8-oxoG, 8-oxoadenine, fapy-guanine, methy-fapy-guanine, fapy-adenine, aflatoxin B1-fapy-guanine, 5-hydroxy-cytosine, and 5-hydroxy-uracil (Hatahet *et al.*, 1994; Tchou *et al.*, 1994). The enzyme OGG1 recognizes only 8-oxoG, and the enzyme Endo III detects oxidized pyrimidines (Kuznetsov *et al.*, 2015). According to our results it is possible to suggest that nicotine was capable of inducing oxidized purines.

According to Aguiar *et al.* (2013), the action of cotinine and nicotine is related to oxidative stress, which can result in DNA lesions, leading to genomic instability and cell death. Guanine is considered the most susceptible base to oxidation due to the low redox potential, with 8-oxoG being considered the most common damage. Regarding oxidative damage, Da Silva *et al.* (2010) have demonstrated an increase in catalase (CAT), which is responsible for decomposing hydrogen peroxide into water and oxygen, in mice exposed to nicotine. Additionally, Kahl *et al.* (2012) demonstrated the potential of vitamin C in reducing the genotoxicity of nicotine. These studies demonstrated that nicotine affects the action of the superoxide dismutase, catalase, and glutathione reductase (Yildiz, 2004), indicating an oxidative stress mechanism in the DNA damage caused by nicotine.

The oxidation process is endogenous and causes considerable DNA damage. The imbalance between the formation and removal of ROS can contribute to degenerative diseases such as cataracts, Parkinson's disease, diabetes, cancer, and aging in general. These highly reactive species can form a wide variety of mutagenic DNA adducts. DNA attacks by ROS can cause the formation of 8-oxo-7,8-dihydro-2’-deoxyguanosine (8-oxodG), which is formed by the hydroxylation of deoxyguanosine in DNA. Chronic exposure to nicotine may increase the production of ROS via the Ras pathway in rats with potential DNA damage and cell cycle deregulation, as well as an increase in nitric oxide (NO) that may play a role in nicotine genotoxicity (Ginzkey *et al.*, 2012). In addition, Feltes *et al.* (2013) using biology systems demonstrated that nicotine may be related to oxidative imbalance of the cell and affect cellular proliferation.

In conclusion, our study indicated that cotinine and nicotine induced cytotoxicity and DNA damage to exposed cells. The similar DNA damage effects observed for these two pyridine alkaloids may be due to the similarity of their structures. The results of the modified comet assay using enzymes demonstrated oxidized purine bases suggesting an oxidizing nature in DNA damage in cells treated with nicotine. In addition, our results demonstrated that cotinine could be also related to the DNA damage observed in individuals exposed to nicotine, and as far as we know, this is the first study on the effect of cotinine on DNA.
